# Foodborne disease control: a transnational challenge.

**DOI:** 10.3201/eid0304.970414

**Published:** 1997

**Authors:** F K Käferstein, Y Motarjemi, D W Bettcher

**Affiliations:** Food Safety Unit World Health Organization, Geneva, Switzerland. Kafersteinf@who.ch

## Abstract

In the globalized political economy of the late 20th century, increasing social, political, and economic interdependence is occurring as a result of the rapid movement of people, images, values, and financial transactions across national borders. Another consequence of the increase in transnational trade, travel, and migration is the greater risk of cross-border transmission of infectious diseases. As the world becomes more interconnected, diseases spread more rapidly and effectively. With more than one million people crossing international borders every day, and with the globalization of food production, manufacturing, and marketing, the risk of infectious disease transmission is greater. Economic globalization has also increased the need for governmental budget austerity, and consequent national preparedness has been eroded. The emergence of new infectious diseases, as well as the reemergence of old ones, thus represents a crucial transnational policy issue. These problems cannot be resolved by national governments alone; they require international cooperation. This article analyzes the role of foodborne disease surveillance programs, nationally and internationally, in the control of foodborne diseases.


### Foodborne Disease Control: A Transnational Challenge

**F. K. Käferstein, Y. Motarjemi, and D. W. Bettcher**
World Health Organization, Geneva, Switzerland

*"Disease knows no boundaries and borders are porous to disease."*(1)

In the globalized political economy of the late 20th century, increasing social, political, and economic interdependence is occurring as a result of the rapid movement of people, images, values, and financial transactions across national borders. Another consequence of the increase in transnational trade, travel, and migration is the greater risk of cross-border transmission of infectious diseases. As the world becomes more interconnected, diseases spread more rapidly and effectively. With more than one million people crossing international borders every day, and with the globalization of food production, manufacturing, and marketing, the risk of infectious disease transmission is greater. Economic globalization has also increased the need for governmental budget austerity, and consequent national preparedness has been eroded. The emergence of new infectious diseases, as well as the reemergence of old ones, thus represents a crucial transnational policy issue. These problems cannot be resolved by national governments alone; they require international cooperation. This article analyzes the role of foodborne disease surveillance programs, nationally and internationally, in the control of foodborne diseases.

In the past two to three decades, public health authorities in industrialized countries have been faced with an increasing number of food safety problems. In 1983, a Joint Food and Agriculture Organization/World Health Organization Expert Committee on Food Safety concluded that illness due to contaminated food was perhaps the most widespread health problem in the contemporary world and an important cause of reduced economic productivity (2). More recent data from industrialized countries indicate that annually up to 10% or more of the population may have a foodborne disease. The situation is equally serious in developing countries, where infant diarrhea causes many illnesses and deaths. In addition to known foodborne diseases, public health communities are being challenged by the emergence of new or newly recognized types of foodborne illnesses, often with serious and chronic health consequences. Certain populations (e.g., pregnant women, the elderly, infants and children, immunocompromised persons, and the undernourished) are particularly vulnerable. In economic terms, foodborne illnesses are very costly for industry, health services, and society as a whole.

Address for correspondence: F.K. Käferstein, Food Safety Unit, World Health Organization, 20 Avenue Appia, CH-1211 Geneva 27, Switzerland; fax: 41-22-791-4807; e-mail: Käfersteinf@who.ch.

Many factors have contributed to the increase in foodborne disease. Industrialization, leading to increased wealth and urbanization, has revolutionized the food supply system, resulting in mass production and an explosive increase in the number of food service establishments and food outlets. Mass production, environmental factors, and inadequate knowledge on the part of food handlers have contributed to increased contamination of primary foodstuffs.

The increase in international trade has increased the risk for cross-border transmission of infectious diseases. The globalization of food (and feed) trade, facilitated by the liberalization of world trade, while offering many benefits and opportunities, also presents new risks (3). Food, a major trade commodity, is also an important vehicle for transmission of infectious diseases. Because food production, manufacturing, and marketing are now global, infectious agents can be disseminated from the original point of processing and packaging to locations thousands of miles away. This multinational approach to food production and distribution and the progressive opening up of world markets have allowed the international food trade to flourish. The value of food trade, U.S. $266 billion in 1994, was more than 300% greater than it was 20 years ago and continues to grow rapidly (4).

The globalization of foodborne diseases also results from increased travel. International travel is more accessible today. The World Tourism Organization estimates world tourist arrivals at 567 million in 1995, and this figure is expected to rise to 660 million by the year 2000. Over the past 200 years, the average distance traveled and the speed of travel have increased 1,000 times while incubation periods for diseases have not changed. As a result, a person can be exposed to a foodborne illness in one country and expose others to the infection in a location thousands of miles from the original source of the infection (5). Depending on their destination, travelers are estimated to run a 20% to 50% risk of contracting a foodborne illness.

As international trade and travel increase, foodborne disease outbreaks of the same origin are more likely to occur in different parts of the globe. Food safety in the late 20th century represents a transnational challenge requiring enhanced levels of international cooperation in setting standards and regulations and in strengthening surveillance systems. Effective food safety programs, built on a clear understanding of the epidemiology of foodborne disease, must be developed and implemented. The globalization of the world's economy has been accompanied by intense economic competition and increased pressure on governments to downsize. Public sector austerity has reduced disease surveillance in many countries (6). For example, in Great Britain, the failure to maintain public health infrastructures has, in the words of the British Medical Association, resulted in "Britain returning to the 19th century in terms of public health, with problems such as dirty water, contaminated food, and old infectious diseases reemerging" (7). Failing a reversal of this trend, public health authorities and health services may be overwhelmed in the near future by outbreaks or epidemics of foodborne diseases. The 1991 epidemic of cholera in Peru and the 1996 outbreak of *Escherichia coli* O157 in Japan demonstrate how one single foodborne disease epidemic or outbreak may disrupt the functioning of a health-care system.

Epidemiologic surveillance of foodborne illness is fundamental to the planning of food safety programs and the development of a strategy for prevention and control. There are different methods of surveillance: death registrations and hospital discharges; disease notification; laboratory-confirmed cases; sentinel surveillance; surveillance of investigated outbreaks; population-based surveillance; and case-control studies of sporadic cases (8). This article examines the role of foodborne disease surveillance programs, nationally and internationally, in the control and prevention of foodborne disease.

### Foodborne Disease Awareness of Public Health Authorities

Data on the incidence of foodborne illnesses collected through notifications, laboratory confirmations, and sentinel or population-based studies can provide a measure of the magnitude of the foodborne disease problems, their economic consequences, and over the years, an indication of the trend. Although several weaknesses are associated with the collection of such data—particularly those collected through notification and laboratory confirmations (since they represent only the tip of the iceberg)—they can nevertheless be useful in raising the awareness of public health authorities about the importance of food safety.

Surveillance data collected in some industrialized countries confirm that foodborne diseases constitute one of the most widespread health problems and that they have increased over the last two or three decades (Figures 1-4). Part of the increase may be attributable to recent improvements in information reporting and collection systems, improved diagnoses, or greater publicity and concern about food safety in general. However, a real increase of foodborne disease incidence is not disputed. First, the increase has been steady and cannot be explained by a one-time improvement in the surveillance system. Second, increases have been observed in different countries, including those with no improvement in reporting and surveillance programs. The general increase, as demonstrated by the results of surveillance data, has led many public health authorities to take stringent regulatory and

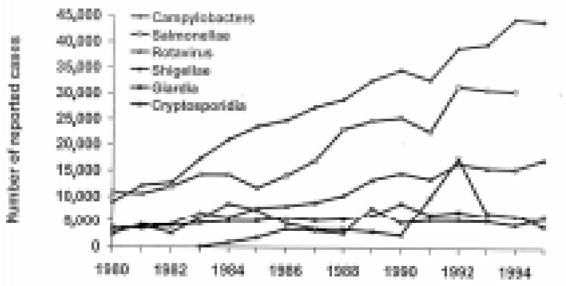

Figure 1. Laboratory reports of gastrointestinal infections in England and Wales.

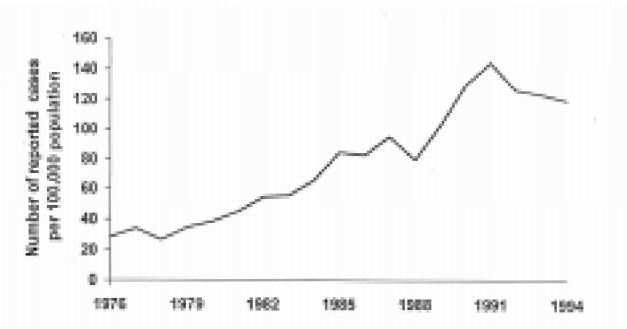

Figure 2. Incidence of foodborne diseases in Venezuela.

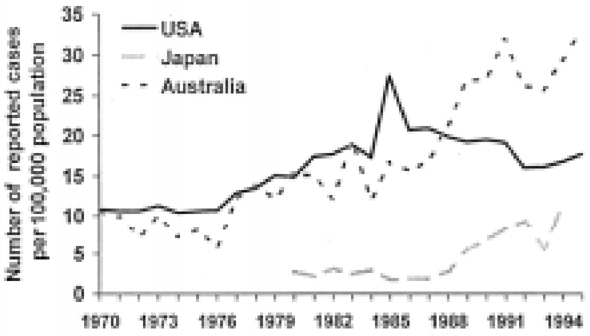

Figure 3. Incidence of salmonellosis in the United States, Japan, and Australia.

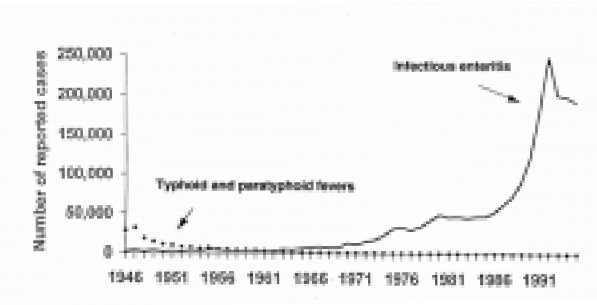

Figure 4. Incidence of infectious enteritis and typhoid and paratyphoid fevers in Germany.

educational measures to improve food safety, with some successful results (9). For instance, in the United States, active surveillance of foodborne listeriosis has led to concerted efforts by industry and government to prevent the disease. As a consequence, the number of cases and deaths has decreased by 44% and 48%, respectively (10).

Public health authorities must be aware of the magnitude and trend of foodborne illness so that necessary resources can be mobilized to improve food safety programs. Lack of reliable epidemiologic data in many parts of the world has impeded the recognition of the public health importance of food safety and consequently the emphasis on food safety programs.

### Early Detection Of Foodborne Disease Outbreaks

Surveillance of foodborne diseases plays an important role in the early detection of foodborne disease outbreaks and their control. Early identification of the source of the outbreak is becoming increasingly important as countries move towards industrialization. Increased mass production means outbreaks can change from being small and confined to a family to large, affecting hundreds or even thousands of people (Table).

Rapid investigation of foodborne disease outbreaks is crucial to prevent them from taking on massive proportions. In the 1993 French outbreak of listeriosis due to potted minced pork (affecting 39 persons and causing eight miscarriages and one death), public health authorities traced its source within 1 week and thus prevented the outbreak from spreading by removing the implicated food product from the market and informing the group at risk about its unsafe nature (11). In an outbreak of botulism in

Table. Examples of large foodborne disease outbreaks

| Country | Year | Disease | No. cases |
|---|---|---|---|
| United Kingdom | 1985 | Salmonellosis | 1,000 |
| United States | 1985 | Salmonellosis | >168,000 |
| United States | 1993 | Salmonellosis | 224,000 |
| China | 1988 | Hepatitis A | >310,000 |
| Germany | 1993 | Salmonellosis | 1,000 |
| Australia | 1991 | Norwalk-like agent | >3,050 |
| United States | 1992-93 | *E. coli* O157 infection | >500 |
| Japan | 1996 | *E. coli* O157 infection | >6,000 |

the United Kingdom traced to hazelnut yogurt, the source was identified within 3 days, and the product was withdrawn from the market (12).

Because of global food distribution and worldwide travel, an international exchange of information on foodborne disease incidences and outbreaks and the foods involved is extremely important to identify international clusters originating from a common source. For instance, Salm-Net, a network for the international surveillance of human salmonellosis, has demonstrated the value of such an interactive international collaboration. Individual countries with apparently isolated outbreaks can feed their information into the network and ascertain whether the outbreak is confined to their country or is of wider international importance. The identification and investigation of several international outbreaks have been simplified by the Salm-Net network.

### Food, the Transmission of Diseases, and the Identification of Associated Risk Factors

Information collected through investigation of foodborne disease outbreaks or case-control studies of sporadic cases provides a better understanding of the role of food in the transmission of communicable diseases and in the identification of risk factors leading to disease. Epidemiologic data from foodborne disease surveillance can provide public health authorities with important information about the types of food implicated in outbreaks; populations at risk; practices that lead to contamination, growth, and survival of foodborne pathogens; and places where foods are often mishandled. Such data are essential for designing effective intervention programs. Such programs in industrialized countries, for example, have demonstrated the relatively greater prevalence and incidence of foodborne diseases of microbial origin over those of chemical origin and the role of food handlers in the transmission of diseases; they have identified campylobacteriosis and salmonellosis (particularly infections caused by *Salmonella* Enteritidis) as the leading foodborne diseases. The emergence of other diseases, such as infections due to *E. coli* O157 and *Listeria monocytogenes*—often with serious sequelae—has been pinpointed as a major public health problem. These surveillance programs have also alerted public health authorities to the foods most often implicated and the major risk factors in food preparation.

Because of the lack of epidemiologic data, the role of food in the transmission of diseases has been poorly acknowledged, particularly in developing countries. Diarrheal diseases in infants and children and diseases such as shigellosis and cholera have been perceived as being water-borne for many years. For instance, after the cholera epidemic in Peru (where epidemiologic investigations implicated, among other foods, seafood, and an embargo was placed on trade in foodstuffs), the role that food plays in the transmission of the disease began to be fully recognized.

Increased trade in food, international travel and migration, and economic and technologic development have changed dietary habits. New foods, food preparations, and dietary habits are introduced into different regions, and as a consequence, foodborne diseases are emerging or reemerging. Dietary habits are also changing as a result of nutritional recommendations and campaigns or may be influenced by food policy, production systems, or environmental changes that lead to increased access to certain foods. These changes in dietary habits influence the epidemiology of foodborne illnesses and contribute to the emergence of foodborne diseases. In the United States, public information campaigns promote an increased consumption of fruits and vegetables. To meet the increased demand, these products have to be imported on a seasonal basis. At certain times of the year, more than 75% of the fresh fruits and vegetables available in grocery stores and restaurants are imported (13). Epidemiologic data have shown that, partly as a consequence of the increased consumption of fruits and vegetables, the proportion of foodborne disease outbreaks has doubled (14).

Data collected through foodborne disease surveillance programs permit the monitoring of changes in the epidemiology of foodborne diseases and the identification of new pathogens and new dietary or food preparation habits that may present a health risk. The data can also determine if existing programs need to be readjusted to ensure that the food safety program is adequate and relevant.

A method used in recent years to complement epidemiologic data in identifying risky practices and behavior is the Hazard Analysis and Critical Control Points system (HACCP). Application of HACCP to food preparation permits the identification of practices that may be potentially hazardous and need to be modified or those that are critical for ensuring the safety of foods and require specific monitoring. However, the first principle of HACCP—to conduct a hazard analysis—calls for epidemiologic data on foodborne diseases, as the process involves an appraisal of the possibility of hazards and the severity of their effects; the qualitative and quantitative evaluation of the presence of hazards; the survival and multiplication of microorganisms of concern; the production or persistence of toxins, chemicals, or physical agents in foods; and, conditions leading to the above.

As demonstrated in the decision tree for hazard analysis (Figure 5) (15), access to information would be difficult without epidemiologic surveillance of foodborne diseases. Similarly, epidemiologic data are also needed to develop sampling plans of food, as demonstrated in the decision tree for *Listeria monocytogenes* sampling plans of foods (Figure 6) (16).

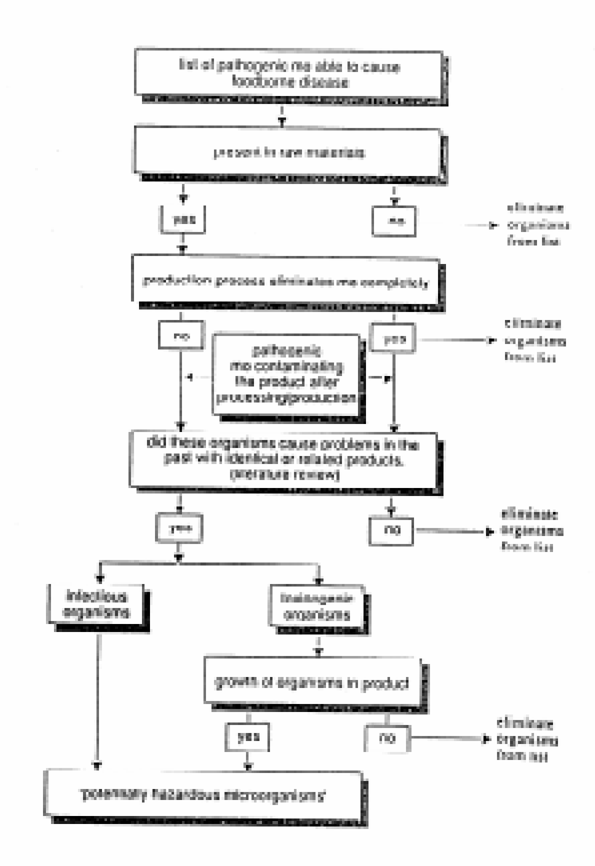

Figure 5. Hazard identification: identification of potentially hazardous microorganisms (15).

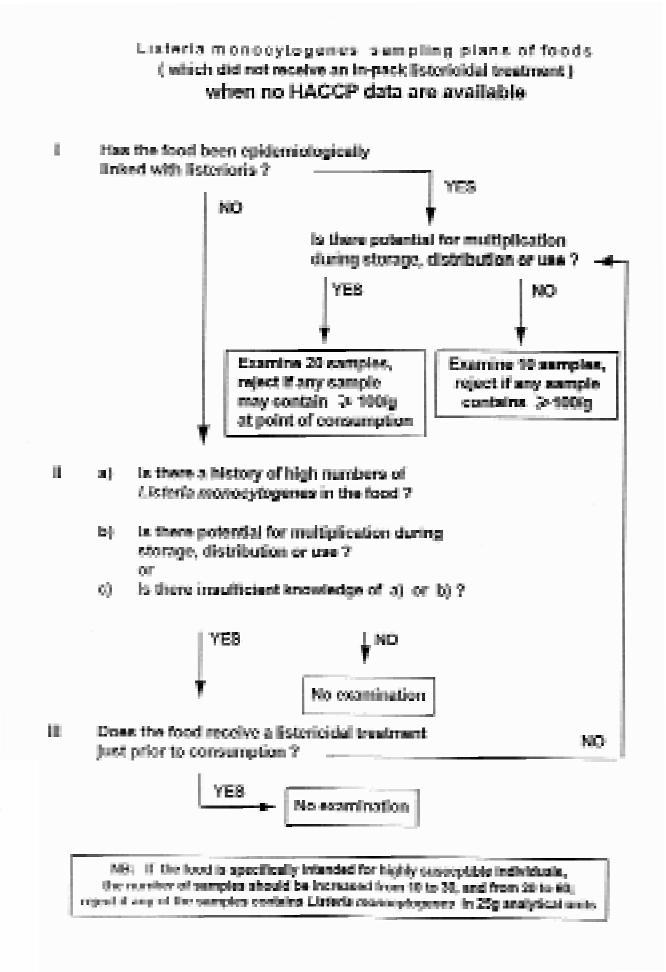

Figure 6. *Listeria monocytogenes* sampling plans of foods that did not receive an in-pack listericidal treatment (16).

### Planning and Evaluating the Effectiveness of Food Safety Programs

The collection of epidemiologic data is important in planning interventions and setting priorities. Countries with scarce resources, facing an abundant number of foodborne diseases and food safety problems, need to prioritize food safety issues. Epidemiologic data provide a basis for identifying foodborne diseases, groups at risk, or even priority points in the food chain.

Evaluating the effectiveness and impact of an intervention is an important element of any plan. Data collected through disease notification or sentinel studies permit an evaluation of the effectiveness of interventions and their impact on health, and if necessary, the adjustment of a program to improve its efficacy and impact. Data on the rising incidence of foodborne illnesses in many countries demonstrate that present prevention strategies, mainly based on regulatory measures, are inadequate and emphasize the need for additional measures (e.g., additional regulatory initiatives and health education about food safety).

### Risk Assessment and International Food Standards

The movement of ever-increasing quantities of food across borders has resulted in a transnationalization of disease risk (17). Therefore, the globalization of food trade and the open access to foreign markets need to be accompanied by effective means of health protection for populations. In the food sector, international regulatory instruments need to be integrated with strengthened surveillance and monitoring.

As a result of the Uruguay Round of Multilateral Trade Negotiations and the increased liberalization of trade facilitated by this agreement, concern about the safety of imported food has grown. However, provisions in the Agreement on the Application of Sanitary and Phytosanitary Measures, which entered into force with the establishment of the World Trade Organization on January 1, 1995, are designed to address these concerns: according to the work of the Codex Alimentarius Commission, its standards, guidelines, and recommendations are recognized as the reference for national food safety requirements. Countries that are members of the World Trade Organization may no longer be able to reject foods that meet Codex standards, guidelines, and recommendations without providing justification.

Moreover, the increased volume of the global food trade underscores the need for sound epidemiologic information and international risk assessment. In this regard, Article 5 of the Sanitary and Phytosanitary Measures agreement explicitly requires World Trade Organization members to conduct scientific and consistent risk assessments. Furthermore, the World Health Organization has recommended that the application of the HACCP system at every stage of the food chain represents an effective approach for governments to meet the terms outlined in the agreement (18).

Another issue receiving more attention from regulatory agencies and underlined during the Food and Agriculture Organization/World Health Organization Conference on Food Standards, Chemicals in Food, and Food Trade (1991), is the scientific basis of the Codex standards. The Conference recommended that the Codex, in its norm-setting work on health and safety, place greater emphasis on risk assessment (19). Epidemiologic data on foodborne diseases have an important role in risk assessment. One example is assessing the risk of contracting listeriosis associated with different levels of *Listeria monocytogenes* in smoked fish and meat products (16). However, the need for risk assessment as the basis for setting standards has shown a great gap in knowledge about foodborne pathogens and their relation to human illness (20-22). To address the national/transnational risks caused by foodborne diseases, this gap must be narrowed.

### Risk Assessment Approach

Risk assessment is defined as a scientifically based process that has the following steps: 1) Hazard identification—The identification of biologic, chemical, and physical agents present in a particular food or group of foods that can cause illness. 2) Hazard characterization—The qualitative or quantitative evaluation of the nature of the illness associated with biologic, chemical, and physical agents that may be present in food. For chemical agents, a dose-response assessment should be performed. For biologic or physical agents, a dose-response assessment should be performed if the data are obtainable. 3) Exposure assessment—The qualitative or quantitative evaluation of the likely intake of biologic, chemical, and physical agents in food as well as exposures from other sources. 4) Risk characterization—The qualitative or quantitative estimation, including uncertainties, of the probability of and severity of known or potential illness in a given population on the basis of hazard identification, hazard characterization, and exposure assessment.

In many cases, data are not available to support a quantitative risk assessment of biologic hazards. We discuss next the types of challenges that make quantitative risk assessment difficult for pathogenic organisms associated with food and the role of epidemiologic surveillance.

### Hazard Identification

Because only some foodborne disease outbreaks are adequately investigated and have the etiologic agents identified, many foodborne pathogens remain unidentified. Most of the available epidemiologic data are furnished by industrialized countries, while the situation in developing countries is largely unknown. The epidemiologic database must be extended to include information from developing countries. However, investigation and surveillance systems in developing countries need to be strengthened before the database can expand.

### Hazard Characterization

For many foodborne pathogens, dose-response data are limited or nonexistent. Information on which dose-response estimates can be based is difficult to obtain and may be inaccurate for various reasons: host susceptibility to pathogens is highly variable, attack rates from a specific pathogen may vary widely, virulence of a pathogenic species is highly variable, pathogenicity is subject to genetic variation resulting from frequent mutation, antagonism from other bacteria in foods or the digestive system may influence pathogenicity, and foods may modulate the ability of bacteria to infect or otherwise affect the host.

### Exposure Assessment

An exposure assessment will give an estimate of either the number of pathogenic organisms or the level of toxins consumed in food. Although the levels of chemical agents in food may change only slightly due to processing, the population of bacterial agents is dynamic and may increase or decrease dramatically. Changes in populations of bacteria are affected by complex interactions of these factors: ecology of the bacterial pathogen; processing, packaging, and storing of food; preparation steps, such as cooking, which may inactivate bacterial agents; and cultural factors relating to consumers.

In addition, for some of the emerging foodborne pathogens, the sources of exposure are still not fully understood. Information on foodborne disease outbreaks provides an opportunity to learn about the types of foods that may harbor the pathogen.

### Risk Characterization

Characterizing the risk associated with biologic pathogens depends on information gained in the previous steps. Risk characterization will result in a qualitative or quantitative estimate of the potential for adverse effects from a particular pathogen on a specific population. Whether a quantitative risk assessment approach is possible and appropriate for characterization of risks associated with foodborne pathogens is not known. Thus, the qualitative approach to characterizing risk may be the only alternative.

### International Travel

International travel and migration are contributing factors in the spread of foodborne diseases in some countries. For instance, 80% to 90% of the incidence of salmonellosis in Scandinavian countries is attributed to international travel. Surveillance of travel-related foodborne diseases provides a mechanism for appreciating the relative prevalence of foodborne diseases in various countries. It also provides a basis for informing physicians and health services about unfamiliar diseases contracted by travelers returning from distant places. In this way, advice on precautionary measures can also be given to travelers. The only foodborne disease now covered by the International Health Regulations is cholera, which is reported to the World Health Organization. Since the purpose of these regulations is to help provide maximum security against the international spread of diseases with a minimum of interference with world traffic (i.e., trade and travel) (23), it is timely to consider whether the regulations should cover additional foodborne diseases.

### Conclusion

The globalization of the risks associated with foodborne illness, specifically increased international travel and trade in food, has resulted in greater interdependence in terms of food safety. Therefore, internationally agreed-upon food safety standards and other types of agreements are becoming increasingly important in addressing the complex transnational challenge of foodborne disease control. Epidemiologic data provide a common ground for reaching international consensus on food safety issues.

As Morris Potter has said, “If one recognizes that ensuring food safety is inherently uncertain, foodborne illnesses become opportunities to learn rather than failures to predict. Foodborne disease will occur, and we must be prepared to react quickly to reduce the risk of new foodborne hazards” (24).

### References

1. Kemel, W. Health dilemma at the borders: a call for global action. In: Proceedings of the 34th Session of the WHO Advisory Committee on Health Research; 1996 Oct; Geneva, Switzerland. Geneva: World Health Organization; 1996.
2. FAO/WHO. The role of food safety in health and development. Report of Joint FAO/WHO Expert Committee on Food Safety. World Health Organ Tech Rep Ser; 1984;705.
3. Fidler D. Globalization, international law and emerging infectious diseases. Emerg Infect Dis 1996;2:77-84.
4. FAO. The world food summit. FAO technical background document no. 12;1996.
5. WHO. International response to epidemics and applications of the International Health Regulations: report of a WHO consultation. Geneva: World Health Organization unpublished document;1996;WHO/EMC/IHR/96.1.
6. Berkelman RL, Bryan RT, Osterholm MT, LeDuc JW, Hughes JM. Infectious disease surveillance: a crumbling foundation. Science 1994:264:368-70.
7. Ferriman A. Doctors warn of a return of past plagues. The Independent 1997 Mar 7; News section:5.
8. Borgdorff MW, Motarjemi Y. Foodborne disease surveillance: What are the options? World Health Stat Quart 1997;50(1/2):12-23.
9. Motarjemi Y, Käferstein FK, Moy G, Miyagawa S, Miyagishima K. Importance of HACCP for public health and development. The role of the World Health Organization. Food Control 1996;7:77-85.
10. Tappero JW, Schuchat A, Deaver KA, Mascola L, Wenger JD. Reduction in the incidence of human listeriosis in the United States. JAMA 1995;273:1118-22.
11. Goulet V. Investigation en cas d'épidemie de listériose, Méd Mal Infect 25 Spécial 1995;184-90.
12. O'Mahony M, Mitchell E, Gilbert RJ, Hutchinson DN, Begg NT, Rodhouse JC, Morris JE. An outbreak of foodborne botulism associated with contaminated hazelnut yoghurt. Epidemiol Infect 1990;104:389-95.
13. Hedberg CW, MacDonald KL, Osterholm MT. Changing epidemiology of food-borne diseases: a Minnesota perspective. Clinical Infect Dis 1994;18:671-80.
14. Wachsmuth K, Kruse H, Tauxe R, Hedberg C, Potter M. Microbial hazards and emerging issues associated with produce. Journal of Food Protection. In press 1997.
15. Notermans S, Mead GC. Incorporation of elements of quantitative risk analysis in the HACCP system. Int J Food Microbiol 1996;30:157-73.
16. Van Schothorst M. Sampling plan for Listeria monocytogenes. Food Control 1996;7:203-8.
17. Nakajima H. Global disease threats and foreign policy. Brown Journal of World Affairs. In press 1997.
18. WTO. Selected World Health Organization activities relevant to the application of sanitary and phytosanitary measures. Geneva: WTO;1995;G/SPS/W/37.
19. FAO/WHO. Report of the Joint FAO/WHO Conference on Food Standards, Chemicals in Food and Food Trade. Rome: FAO;1991.
20. FAO/WHO. Report of the Joint FAO/WHO Expert Consultation on Application of Risk Analysis to Food Standards Issues, Geneva, 1995 13-17 Mar. Geneva: World Health Organization, unpublished document;1995;WHO/FNU/FOS/95.3.
21. FAO/WHO. Report of the Joint FAO/WHO Expert Consultation on Risk Management and Food Safety Issues, Rome, 1997 27-31 Jan. FAO Food Nutr Pap 1997;62.
22. Bernard DT, Scott VN. Risk assessment and foodborne micro-organisms: the difficulties of biological diversity. Food Control 1995;6:329-33.
23. WHO. International Sanitary Regulations: World Health Regulation No. 2. Geneva: World Health Organization;1951.
24. Potter M, Gonzalez-Ayala S, Silarug N. The epidemiology of foodborne diseases. In: Doyle MP, Beuchat L, Montville T, editors. Fundamentals of Food Microbiology. Washington (DC): American Society for Microbiology; 1997.
